# Perspective: What is the Likely Impact of the Proposed Food and Drug Administration Front-of-Package Criteria on the Labeling of Foods?

**DOI:** 10.1016/j.cdnut.2026.107638

**Published:** 2026-01-16

**Authors:** Paula R Trumbo, Adam Drewnowski

**Affiliations:** 1 Paula R. Trumbo Consulting, Mount Pleasant, SC, United States; 2 Department of Public Health, School of Health Sciences, Liberty University, Lynchburg, VA, United States; 3 Department of Epidemiology, Center for Public Health Nutrition, University of Washington, Seattle, WA, United States

**Keywords:** front-of-package labeling, sodium, saturated fat, added sugars, dietary fiber, calcium

## Abstract

The United States Food and Drug Administration (FDA) recently proposed a new rule on front-of-package (FOP) labeling. Focusing on added sugars, sodium, and saturated fat, the proposed FOP label would identify foods with low [<5% daily value (DV)], medium (6‒19% DV), and high (20% DV) concentrations of each nutrient, calculated per serving size. This study applied the proposed FDA criteria to food categories and subgroups as defined in the USDA Food and Nutrient Database for Dietary Studies (2017‒2018). Nutrient composition data were joined with the FDA Reference Amounts Customarily Consumed. Analyses show the distribution of foods into FDA low, medium, and high criteria for each nutrient. Further analyses addressed food categories that were good (10%‒19% DV) or high/excellent (20% DV) sources of calcium, protein, and dietary fiber. This proposed labeling approach focuses only on nutrients to limit, similar to “warning label” FOP approaches seen in other countries. A balanced labeling approach that also includes nutrients to encourage (e.g., protein, dietary fiber, vitamins, and minerals) may be the preferred option to establish the relative healthfulness of foods.

**Statement of Significance**

Front-of-package (FOP) labeling is meant to provide an indication of the healthfulness of a packaged food product to the consumer. Healthfulness is often viewed as the absence of added sugars, sodium, or saturated fat. This study is the first to apply the proposed Food and Drug Administration criteria to foods in the What We Eat in America nutrient composition database. The distribution into low, medium, and high FOP classes is provided for each of the 3 nutrients to limit by food category. Analyses were also conducted for dietary fiber, calcium, and protein. The findings include the following, as expected; meats contain no added sugars whereas beverages are low in saturated fat. The healthfulness of a food is not solely determined by nutrients to limit, a more balanced approach is preferred. With the provision of only nutrients to limit, the consumers could be misled and misinformed about the overall healthfulness of a food.

## Introduction

Front-of-package (FOP) labeling is meant to indicate the healthfulness of a packaged food product [1]. The FOP Nutrition Info box, recently proposed by the Food and Drug Administration (FDA) [1], will indicate whether a given food contains low [<5% daily value (DV)], medium (6%‒19% DV), or high (>20% DV) amounts of saturated fat, sodium, and added sugars per serving. The FDA proposal would not prohibit additional voluntary claims about nutrients to encourage, only standardize the FOP presentation of the nutrients to limit. Current FDA regulations already permit nutrient content claims for protein, dietary fiber, vitamins, and minerals. The proposed FOP percentages are somewhat aligned with the existing definitions for “low” (∼<5% DV), “good” (10%‒19% DV), and “high” (>20% DV) sources of a given nutrient that are used in nutrient content claims [2,3].

The focus on the 3 nutrients to limit perpetuates the concept of negative nutrition. The healthfulness of foods is viewed by policy-makers as the absence of calories, fat, sugar, and salt, and with less emphasis on the beneficial nutrients that a food may actually contain [4]. To substantiate this position, the FDA published a 2023 literature review that includes 65 original studies and surveys that explore 4 key global FOP labeling systems (Nutri-Score, Health Star Rating, Warning Signs, and Multiple Traffic Lights) and their impact on consumer food choices [5]. To differing extents, those FOP systems also focus on calories and on the nutrients to limit: total or added sugars, sodium, and saturated fat. The emphasis on negative nutrition is not unique to the FDA.

The proposed FOP rule includes a voluntary option for listing calories per serving near the Nutrition Info box. However, the FDA has not provided an option to include nutrients to encourage, such as protein, dietary fiber, or calcium. The proposed Nutrition Info box differs from nutrient content claim regulations in that, for the first time, there would be FOP information on a “medium” or “high” amount of a nutrient to limit. Current regulations only provide for nutrient content claims about a “low” source of nutrients to limit.

FDA has previously considered the potential use of FOP nutrition labeling under the Nutrition Labeling and Education Act and the Food, Drug, and Cosmetic Act authorities. These activities have included public meetings and consumer studies. In 2011, the FDA issued a letter to the Grocery Manufacturers Association (now the Consumer Brands Association) and the Food Marketing Institute (now the Food Industry Association) announcing the agency’s intent to exercise enforcement discretion with respect to certain FDA nutrition labeling regulations so that the associations could introduce and use their Facts Up Front FOP nutrition labeling program [6]. FDA recognized in the letter that the standardized, non-selective presentation of calories, saturated fat, sodium, and total sugar content on a company’s entire product line, if widely adopted by the food industry in a uniform manner, could contribute to FDA’s public health goals by fostering awareness of the nutrient content of foods in the marketplace and helping consumers to make quick, informed, and healthy food choices. The optional inclusion of nutrients to encourage was also permitted.

Different FDA criteria apply to beneficial nutrients or nutrients to encourage. In 1993, when considering the appropriate definition for the nutrient content claim “high” to be >20% DV for a nutrient to encourage, the FDA evaluated its food composition database to examine the types of foods that contain nutrients at levels that are ≥20% of the proposed reference value per serving [7]. It was determined that for the majority of the 17 nutrients to encourage (e.g., vitamins and minerals) considered, at least 10% of the foods in the database contained ≥20% of the DV. For these nutrients, there was at least 1 and often >1 food category that contained a substantial number of foods containing ≥20% of the DV, qualifying them for high claims. Therefore, the agency concluded that the 20% eligibility level would permit a sufficient number of food items to bear a “high” claim to allow consumers to use the claim in selecting a varied diet. The agency also noted that this level provided an appropriate basis for upper-level nutrient content claims that can be readily used by consumers to implement current dietary guidelines [7].

There are several issues that need to be considered in the FDA’s newly proposed FOP labeling. First, the proposal will reinforce to consumers that the absence of nutrients to limit is the only lens by which to determine the healthfulness of a food. In that view, the best foods are those without calories, saturated fat, added sugars, or sodium. Taking the view to its logical extreme, plain water could be (and was) considered a nutrient-dense food. The Dietary Guidelines for Americans 2025 listed sparkling water as an example of a nutrient-dense food, despite the absence of any nutrients at all [8]. It would be more logical to assess the healthfulness of foods using metrics that also incorporate nutrients to encourage (protein, dietary fiber, vitamins, and minerals). Since 1993, regulations have provided for voluntary labeling of content claims for nutrients to encourage (e.g., good or excellent source of calcium), as well as to nutrients to limit (e.g., low). Regulations are not provided for the labeling of “low” for nutrients to encourage, and therefore are prohibited. As such, it is unclear how a consumer would interpret a label that includes both a claim about a food that is “medium” in a nutrient to limit and a “good source” for a nutrient to encourage.

Second, the exclusive focus on added sugars, saturated fat, and sodium as the principal indices of healthfulness seems relatively crude. The recent WHO/FAO joint statement on healthy diets made a valuable point that the moderation criteria (i.e., limits on fat, added sugars, and salt) are applied to total diets and not necessarily to individual foods [9]. Added sugars are metabolically indistinguishable from naturally occurring sugars. The causal role of added sugars, unique from natural sugars, in chronic disease risk is lacking. The observed links between saturated fat and health outcomes can depend on the food matrix and the source and type of saturated fat (e.g., dairy or red meat), and the issue has not been resolved [10]. The current recommendation not to exceed 2300 mg of sodium per day [8] is for overall daily intake in recognition that foods of varying sodium contents across different food groups are part of a healthy dietary pattern. There are existing nutrient profiling methods that take added sugars, saturated fat, and sodium into account and are discussed in detail below.

Third, the FDA argues that the Nutrition Info box will improve consumer nutrition literacy, encourage healthier purchases, and pressure the industry to reformulate its products to be healthier [1]. Indeed, 1 of the key intents behind FOP labels is to encourage the food industry to reformulate.

There are documented examples of European Union companies reformulating products to be more consistent with Nutri-Score [11]. Along the same lines, the FDA has issued phase II voluntary sodium reduction targets in draft guidance, voluntary sodium reduction goals: target mean and upper bound concentrations for sodium in commercially processed, packaged, and prepared foods (edition 2) [12]. The new draft targets, issued on 15 August, 2024, build on the final, voluntary sodium reduction goals issued in 2021 (phase I). Arguably, the proposed FOP labels targeting sodium in foods are 1 way to ensure industry compliance.

Finally, data on consumer behavior from markets where similar FOP systems are in place are less clear. Most studies were based on virtual supermarkets and intent to purchase rather than on purchase behavior [13]. Behavioral nutrition needs to be supplemented by economics of food choice [14]; that healthier food purchases cost more (a formerly controversial topic) is now well established [15]. Pressuring the industry to reformulate does not always lead to expected outcomes.

Where does that leave the FDA proposal? This Perspective paper provides the needed FDA regulatory history along with some valid considerations for a useful FOP label. Data are provided to illustrate how the proposed FOP criteria, calculated per serving, would apply to different food categories in the United States food supply. Those calculations were based on publicly available USDA nutrient composition data, using the FoodData Central [16].

## Methods

### Food and nutrient database for dietary studies 2017‒2018

The What We Eat in America (WWEIA) study is the dietary component of the nationally representative NHANES [17]. A nutrient composition database, known as the USDA Food and Nutrient Database for Dietary Studies (FNDDS) [18], was used to calculate energy and nutrient content of the NHANES participants’ diets. Unlike other USDA databases, the FNDDS contains nutrient data for foods as consumed, allowing for a direct link with American eating habits and consumption patterns, diet quality, and associated health outcomes.

Individual food items in the FNDDS (identified by 8-digit codes) were aggregated by the USDA into food groups, food subgroups, and food categories using WWEIA 1-digit, 2-digit, and 4-digit codes (Table 1). The 2017–2018 FNDDS contains 7083 foods and beverages, aggregated into 16 food groups, 49 food subgroups, and 167 food categories. The FNDDS 2017‒2018 is publicly available and can be downloaded from the USDA FoodData Central. The present analyses used selected FNDDS 2017‒2018 food subgroups (2-digit WWEIA codes), as defined by the USDA. Infant foods, mixed foods, alcohols, and coffee and tea were excluded.

**TABLE 1:** USDA food and nutrient database for dietary studies 2017‒2018 food subgroups used in the present analyses

Food subgroup	No. of items	Food subgroup	No. of items
Milk	29	Crackers	62
Flavored milk	66	Quick breads	126
Yogurt	31	Ready-to-eat cereals	134
Cheese	73	Savory snacks	141
Dairy drinks and soy	40	Snack/meal bars	46
Eggs	151	Sweet bakery product	357
Meats	224	Candy	134
Poultry	224	Fruits	118
Seafood	434	Vegetables	478
Cured meats/poultry	119	White potatoes	149
Plant-based protein	155	100% juice	46
Breads	198	Sweetened beverages	69
Cooked cereals	111	Diet beverages	31
Cooked grains	55	Total	3801

Abbreviation: USDA, United States Department of Agriculture.

The proposed FDA criteria for FOP labeling were based on percent DVs calculated per serving size [retail serving sizes are defined by the FDA Reference Amounts Customarily Consumed (RACC)] [1]. FDA RACC values were manually attached to FNDDS food items in each category. All RACC values were in grams. Beverages with RACC values in milliliters were converted to grams (1 g = 1 mL). Foods that could not be matched with RACC values were excluded.

Analyses focused on the 3 nutrients in the proposed Nutrition Info box: added sugars, saturated fat, and sodium. The percent distribution into low, medium, and high FOP labels for each nutrient, according to the FDA’s proposed criteria and food category, is provided in Table 2. Additional analyses were conducted for protein, calcium, and dietary fiber, nutrients currently listed on the nutrition facts label. For these 3 nutrients, the criteria were “good” and “excellent” sources of the nutrient as defined by the FDA [2,3]. Foods that meet the “good source” claim provide 10% to 19.99% of the DV per RACC; whereas an “excellent source” food provides >20%. Statistical analyses used Microsoft Excel (version 16.66; Microsoft Corporation) and Statistics Package for the Social Sciences v31 from IBM (version 16.0).

**TABLE 2:** Percent daily values and amounts of added sugar, saturated fat, and sodium per reference amount customarily consumed in each proposed Food and Drug Administration category of low, medium, and high

DV, %	Added sugar, g	Saturated fat, g	Sodium, mg
	DV = 50	DV = 20	DV = 2300
Low: ≤5.49	<2.75	<1.1	126
Medium: 5.5‒19.49	2.75‒9.75	1.1–3.9	126‒448
High: ≥19.5	>9.75	>3.9	>448

Abbreviation: DV, daily value.

## Results

### Added sugars, saturated fat, and sodium – low, medium, or high?

**FIGURE 1:**
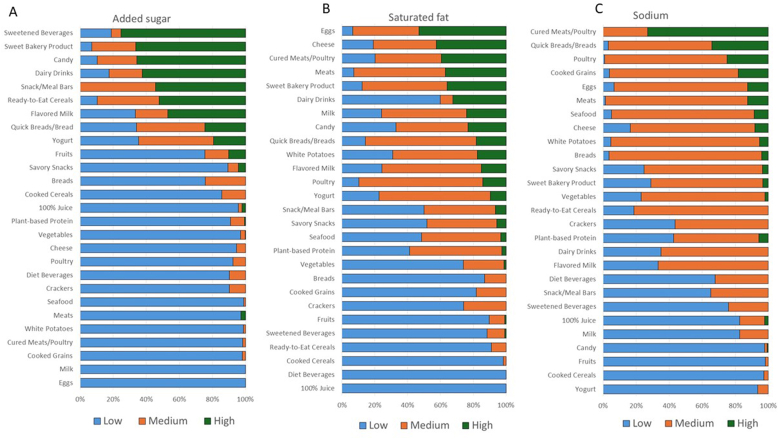
Percent of foods within each food category that meet the FDA proposed criteria for low, medium, and high for added sugars (A), saturated fat (B), and sodium (C). FDA, Food and Drug Administration.

Contrary to popular belief that added sugars are found in many foods, most foods in the United States food supply do not actually contain any added sugars. From our assessment, the number of food categories with added sugars was relatively low; they were found primarily in sweetened beverages, sweet bakery products, candy, dairy drinks and subs, snack/meal bars, ready-to-eat cereals, and flavored milks (Figure 1A). Using FDA’s proposed criteria most (18/27) food categories had a substantial portion (>70%) of foods that were “low” in added sugars, few categories had foods that met the “medium” criteria and only a few categories (most sweet bakery products, candy, and sweetened beverages) were predominantly “high” in added sugars (Figure 1A). This skewed distribution of mostly high or low designations demonstrates the challenges consumers will be faced with when considering what would be a better choice in particular food categories in the absence of options that fall into “medium.”

When foods are high in 1 nutrient to limit, they are often low in the others. For example, foods that contain no added sugars often contain saturated fat and vice versa (chocolate candy is 1 exception; sweet bakery goods are another). Figure 1B shows that eggs, cheese, meat, and processed meat categories had the highest percentages of food items in the FDA’s proposed “high” category for saturated fat. Many categories (e.g., vegetable, fruit) were low in saturated fat. Only a few of the food categories (5/27) included >70% of foods that were high in saturated fat. In other words, for saturated fat, most foods may display a low or medium in the Nutrition Info box, and few would display high. It would be important to understand whether the proposed FOP scheme provides for enough variation in products as substitutes to allow for a meaningful improvement in choices.

Figure 1C shows the distribution of foods across the low, medium, and high criteria for sodium. Most likely to be high in sodium were cured meats, poultry, quick breads, eggs, meats, cooked grains, seafood, and cheese. Candy, fruits, fruit juices, and cooked cereals were low in sodium. Only 6 of 27 categories we examined had ≤10% of foods that were high in sodium. Relatively few sodium-containing foods in this data set were low or high in sodium when using the FDA’s proposed criteria. As a result, most foods fell within the “medium” sodium category, which contrasts with the distribution for added sugars and saturated fat. The usefulness to consumers of having the majority of foods categorized as medium in sodium, or any nutrient to limit, is unclear; is a food that is medium in sodium to be encouraged or discouraged? Including nutrients to encourage would provide additional context for consumers to consider when making a choice.

### Is low-low-low the best option?

**FIGURE 2:**
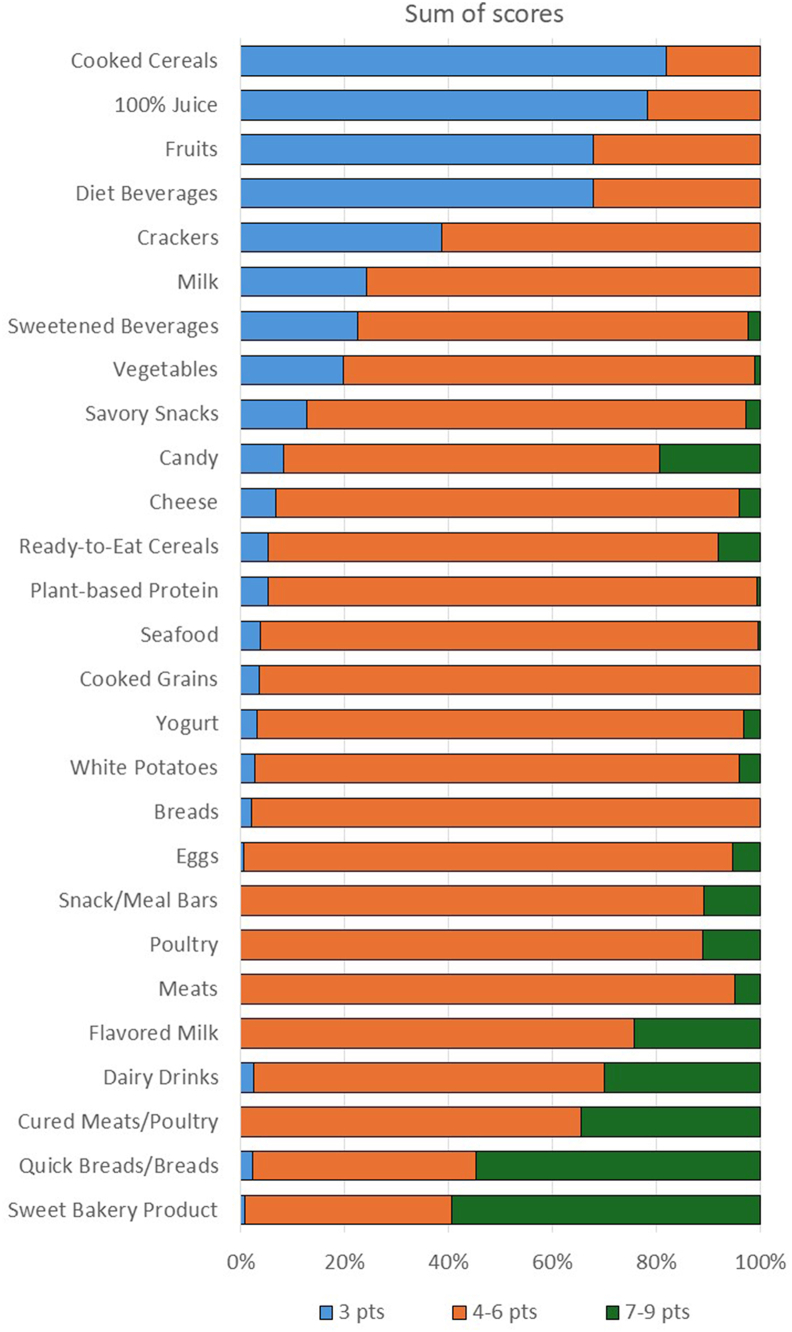
Percent of foods within each food category that meet the FDA proposed criteria for low, medium, and high for the sum of added sugars, saturated fat, and sodium. FDA, Food and Drug Administration; Pts, points.

It is a simple calculation to identify those food categories that score low-low-low using FDA proposed criteria (Table 2). The top 4 healthiest food categories, as defined by the FDA FOP proposal criteria of low-low-low, were cooked cereals, 100% juice, fruits, and diet beverages (Figure 2). Of these 4 food categories, the majority of foods in each were low for all 3 nutrients to limit (diet beverages, cooked cereal, fruit juice, and fruits). The sweet bakery products category was unique in that higher proportions of foods met the medium and high criteria. The majority of food categories had <20% of foods that were low-low-low, therefore limiting food choices and making it difficult to consume certain important low-low-low foods, including meats, bread, seafood, and ready-to-eat cereals. Is the proposed FDA scheme telling us anything that is new and that we do not already know? In our assessment, there were no foods that were “the best” or “the worst” choice; just many foods that were a mix of low, medium, and high designations. Without the addition of nutrients to encourage to provide additional context of the food’s nutritional value to consumers, it may be difficult for them to make a “better,” more nutrient-dense choice.

### The case for positive nutrition – dietary fiber, calcium, and protein

Although the scientific evidence is strong for nutrients to limit (e.g., saturated fat and sodium) and chronic disease risk, the evidence is equally strong for nutrients to encourage, such as dietary fiber, calcium, and protein [19,20]. Thus, including both nutrients to limit and nutrients to encourage in FOP labeling may provide the consumer a better understanding of the overall healthfulness and nutrient density of a food.

### Dietary fiber

**FIGURE 3:**
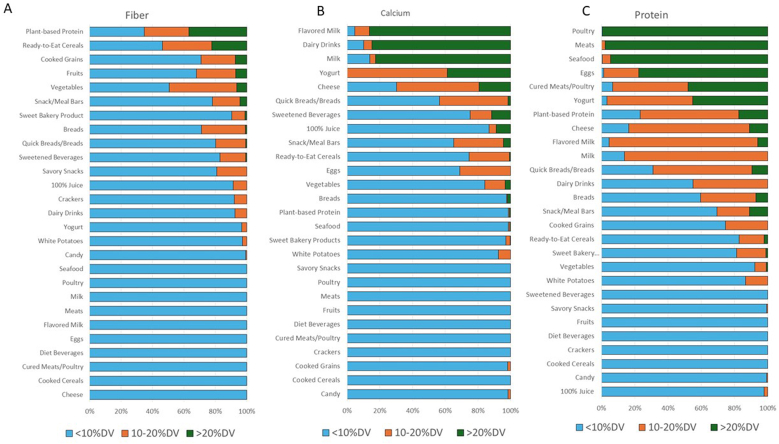
Percent of foods within each food category that meet the criteria for low, good, and excellent sources for dietary fiber (A), calcium (B), and protein (C). DV, daily value.

The top 2 food categories that were major sources of dietary fiber were ready-to-eat cereals and plant-based proteins (e.g., legumes, nuts, and seeds) (Figure 3A). These 2 categories were the only categories to include at least 10% of foods that are high in dietary fiber. The majority (22/27) of categories had foods that were predominantly (>70%) low (i.e., did not meet 10% DV) in dietary fiber. However, ready-to-eat cereals and plant-based proteins would appear to be less healthy than sugar-sweetened beverages and foods when only considering the 3 nutrients to limit (Figure 2).

### Calcium

The 5 top sources of calcium were flavored milk, dairy drinks, milk, yogurt, and cheese (Figure 3B). The majority (19/27) of the food categories contain foods (>70%) that are low in calcium. Flavored milk was the top source of calcium; however, flavored milk was the fourth least healthy product (i.e., 24/27) when only considering the 3 nutrients to limit.

### Protein

The top sources of protein per serving (Figure 3C) were poultry, meats, seafood, eggs, cured meats, and yogurt, followed by plant proteins and cheese. However, many foods in those categories were also high in sodium and saturated fat but low in added sugars. Nutrient density of foods is not fully captured by nutrients to limit only.

### FDA’s proposed approach is confusing and doesn’t align with nutrient content claims

Of further note, the FDA’s proposed FOP label criteria do not align with their newly finalized “healthy” nutrient content claim [21]. For example, foods with small serving sizes, including salty snacks like potato and tortilla chips, will list “low, low, low” FOP designations for all nutrients to limit. They may appear to be a “healthy” choice, even though they would not qualify for the healthy claim. On the other hand, foods that fall into the FDA’s criteria for meals and main dishes can qualify for the “healthy” claim, and list “high” FOP designations for any of the nutrients to limit [21]. If a product were to have a “healthy” claim and display high for nutrients to limit, it could be confusing for consumers.

## Discussion

Consistent with 1993 FDA criteria for “high” [7], our analyses showed that several food categories contained foods with >20% DV per serving of added sugars, sodium, and/or saturated fat. The majority of food categories did not contain any added sugars. The sodium thresholds were such that most foods fell into the medium class. Animal products contained saturated fat; plant-based products generally did not.

A more important consideration, however, is whether providing FOP label information only on saturated fat, sodium, and added sugars is sufficient to inform the consumer about the healthfulness of a food as part of a dietary pattern. The evidence that is provided here clearly suggests that only providing 3 nutrients to limit is insufficient. The top healthiest foods, as identified by the FOP proposal of low-low-low, based on the level or absence of the 3 nutrients to limit, were cooked cereals, 100% juice, fruits, diet beverages, and crackers. Diet beverages and crackers contain few or none of the 3 nutrients to limit, but also contain little or no nutrients to encourage, such as calcium, protein, or dietary fiber. Sweetened beverages and foods (e.g., candy) are discouraged from consumption, but the evidence from this analysis shows that these food categories are equally or more healthy than many categories as defined by the FOP criteria, including plant-based protein, vegetables, eggs, meats, poultry, seafood, cooked grains, and ready-to-eat cereals that are encouraged to consume [8]. Most cooked cereals and 100% fruit juices categories have few or no nutrients to limit, but also are predominantly low in protein, calcium, and dietary fiber. Cheese is 1 of the few categories that provide calcium and protein; however, cheese products tend to be high in sodium for food preservation and safety purposes. Many cereals contain dietary fiber, but some are also high in added sugars, as defined by this FOP proposal.

It is clearly important to include nutrients to encourage, such as dietary fiber and calcium, in any FOP label the FDA is considering. Many nutrient profiling models that balance both nutrients to encourage and nutrients to limit have been designed to do just that [[22], [23], [24]].

Because the intent of the FOP label is to help inform consumers about the healthfulness of foods, such information should be science-based. Although there is evidence for saturated fat and sodium in heart disease risk [25,26], causal evidence for added sugars is lacking for chronic diseases in general [27]. Added sugars are ingredients and are no different than sugars naturally present in foods. The causal evidence for sugars (natural or added) is lacking for the risk of chronic diseases, except for dental caries. The causal evidence for calcium and osteoporosis and dietary fiber and heart disease is well established, and both have health claims for food labeling [28,29]. As such, the evidence for calcium and dietary fiber is significantly stronger than for added sugars. Because of their public health importance and because Americans consume inadequate amounts, dietary fiber and calcium are required to be listed on the nutrition facts label. Such information should, at a minimum, be optional in the Nutrition Info box.

To support the inclusion of only nutrients to limit in the Nutrition Info box, the FDA argues that while many consumers use and benefit from the nutrition facts label, fewer people use the nutrition facts label to look at nutrients to limit (including sodium, saturated fat, and added sugars) in the label [1]. The proposed rule cited results from a 2019 FDA survey [30] that reported that <50% of consumers look at sodium (38%), saturated fat (30%), and added sugars (34%). However, the survey also reported that only 26% of consumers look for dietary fiber and vitamins and minerals. Based on this FDA survey, a smaller percentage of consumers look at the beneficial nutrients (26%) than the nutrients to limit (30%‒38%). Thus, although less than half of consumers look at the nutrients to limit, even fewer look at the positive nutrients.

The 2019 FDA survey [30] also reported that the decision to purchase a product that stated high fiber (42%) on the FOP label was greater than a product labeled as low saturated fat (29%). Calcium and dietary fiber are required to be listed in the nutrition facts label because Americans consume less than what is recommended and because they play an important role in reducing the risk of chronic disease (e.g., cardiovascular disease and osteoporosis). As such, the information in our analysis and consumer studies suggests that including nutrients such as calcium and dietary fiber on the FOP label would provide an overall balance in the healthfulness of a food, reduce consumer confusion, and assist consumers in maintaining healthy dietary practices.

The proposed rule states that, through a focus group, participants were confused by the inclusion of both nutrients to limit and nutrients to encourage in the same FOP scheme [1]. Although the FDA qualitative and quantitative studies tested the combination of negative and positive nutrients, the findings were specific to confusion about the colors used as part of the scheme, according to the FDA consumer studies report [31]. Specifically, it states that participants were confused by the use of the colors red, yellow, and green when schemes contained both nutrients to limit and nutrients to get enough of (e.g., they had trouble interpreting the scheme when red indicated a high amount of a nutrient to limit and a low amount of a nutrient to encourage). As such, no findings were presented on the effectiveness of schemes that included only-negative nutrients, positive nutrients, or a combination. The only conclusions presented were that consumers were confused when using colors [31]. Therefore, the FDA’s studies did not specifically address the issue of which option is most effective in choosing healthy food products. Studies are lacking that directly compare the listing of only nutrients to limit, only nutrients to encourage, and the listing of both while controlling for other influential aspects such as color, shape, and size. Based on a recent online experimental study of ∼3000 United States adults, FOP labels with both-positive-and-negative nutrients outperformed (led to healthier products) FOP labels with only-negative and only-positive-labels FOP labels [32]. A survey in 962 United States adults showed that FOP labeling with nutrients to encourage resulted in the selection of healthier foods compared with FOP labeling that focused on the avoidance of nutrients to limit [33]. Results from a 2024 International Food Information Council consumer study support these findings [34]. Their results suggested that FOP schemes including information, such as dietary fiber, in addition to nutrients to limit, may facilitate consumers in selecting the “healthiest” FOP label.

According to NHANES data from 2017 to 2020, ∼80% of adults reported using the nutrition facts label either sometimes, most of the time, or always when buying packaged food products [35]. The nutrition facts label provides important information on calories, and the most important nutrients to limit and to encourage to assist consumers in maintaining healthy dietary practices. The NHANES data also showed that regular use of the nutrition facts label is associated with lower intake of energy, fat, saturated fat, sugars, and sodium [35]. The FOP proposed rule argues that the Nutrition Info box is intended to complement the nutrition facts label; however, several pieces of evidence showed that FOP labeling can reduce and take away attention from the more comprehensive nutrition facts label [36,37].

Evidence is lacking to suggest that FOP labeling is a material fact and is necessary for consumers to maintain healthy dietary practices. The 2021 International Food Information Council survey on FOP labeling reported that 54% of consumers stated that FOP labeling had an impact on their food and beverage purchases. The impact of the nutrition facts label on food and beverage purchases was 61%. This impact was “significant” for 24% of consumers using FOP labeling, whereas the impact was “significant” for 27% when using the nutrition facts label [38]. Based on a review of the literature on the effects of FOP labeling, it was concluded that *1*) the magnitude of improvements in food choices is small, *2*) there is limited evidence to show that FOP labeling affects reformulation, *3*) evidence of change in negative nutrient intake is inconsistent across different FOP schemes, and *4*) there is insufficient evidence that FOP labeling leads to meaningful improvements in consumer behavior and nutritional quality of packaged foods [13]. Similar conclusions were made by the FDA as a result of their FOP labeling literature review [5].

In summary, the need for FOP labeling is not well substantiated and therefore is questionable. Requiring only sodium, saturated fat, and added sugars in FOP labeling does not necessarily result in informing the consumer about the healthiest foods. In fact, only providing these nutrients to limit could misinform the consumer and result in the selection of less healthy foods than what the FOP label implies. On the contrary, the nutrition facts label is mandatory and provides more comprehensive nutrition information than the proposed FOP labeling scheme. Consumption of healthy foods does not only pertain to nutrients to limit, but also nutrients to encourage, where there is well-established evidence and recommended by the Dietary Guidelines for Americans [8].

## Author contributions

The authors’ responsibilities were as follows – AD: conducted the data analysis; PRT, AD: wrote the paper; PRT: had primary responsibility for final content. Both authors read and approved the final manuscript.

## Funding

This work was supported by the General Mills’ Bell Institute of Health and Nutrition. PRT received funding to draft the manuscript and serve as the corresponding author. AD received funding to conduct the data analysis and draft the manuscript.

## Conflict of interest

PRT has served as a consultant to KraftHeinz, General Mills, Flowers Foods, Ultima, Givaudan, PepsiCo, Johnson & Johnson, Nestlé USA, Ocean Spray, GlaxoSmithKline, Tate & Lyle, Ingredion, Bioneutra, Lantmännen, Hayashibara, MycoTechnology, Quebec Maple Syrup Producers, Colgate Palmolive, Almond Board of California, Constellation Brands, Kappa Biosciences, Kodiak Cakes, Bay State Milling, Intertek, The Protein Brewery, 8Greens, GRAS Associates, ILSI North America, and Institute for the Advancement of Food and Nutrition Sciences. AD is the original developer of the Naturally Nutrient Rich and the Nutrient Rich Food nutrient profiling models and is or has been a member of scientific advisory panels for BEL, Lesaffre, Nestlé, Friesland Campina, National Pork Board, and Carbohydrate Quality Panel supported by Potatoes USA. AD has worked with Ajinomoto, Ayanabio, DSM-Firmenich, FoodMinds, General Mills, KraftHeinz, Meiji, MS-Nutrition, Nutrition Impact LLC, Nutrition Institute, PepsiCo, Samsung, and Soremantec on quantitative ways to assess the nutrient density of foods.
